# Culturally Competent Assessment of Neurocognitive Functioning in Latinos with Complex Multimorbidity: A Case Study

**DOI:** 10.3390/geriatrics7050093

**Published:** 2022-09-06

**Authors:** Diomaris Safi, Jesús Barreto Abrams, Melissa Rios, Elisenda Rodés, Mirella Díaz-Santos, Paola Suárez

**Affiliations:** 1 Department of Psychiatry and Behavioral Sciences, UCLA Hispanic Neuropsychiatric Center of Excellence, 760 Westwood Plaza, C8-238, Los Angeles, CA 90095, USA; 2 California School of Professional Psychology, Alliant International University, Alhambra, CA 91803, USA; 3 Graduate School of Education and Psychology, Pepperdine University, Los Angeles, CA 90045, USA; 4 Department of Neurology, UCLA Mary S. Easton Center for Alzheimer’s Disease Research, Los Angeles, CA 90095, USA

**Keywords:** multimorbidity, cognitive impairment, bilingualism, neuropsychological assessment, cultural competency

## Abstract

Multimorbidity—the coexistence of multiple chronic conditions within an individual—is the new normal in hospital settings. Individuals with higher levels of multimorbidity require a multidisciplinary and holistic approach to meet their needs, though the complexity of their neurocognitive profiles is still poorly researched. This study reported on the neurocognitive profile of a 69-year-old, left-handed, Latino cisgender male with 10 years of education. He was deemed to have a short-term mortality in 2018, yet is still enjoying a good quality of life in 2022. This case report illustrated (a) a rather common neurocognitive profile of a patient with complex multimorbidity, (b) the advantages of being served in a center of excellence with linguistically and culturally appropriate services that evaluate patients’ cognitive functioning and inform and provide continuity of care, and (c) the benefits of a holistic and multidisciplinary approach to the care of the multimorbidity population.

## 1. Introduction

As life expectancies increase, co-occurring chronic health conditions are simultaneously rising in prevalence worldwide [1]. Approximately 60% of individuals ages 65 and older have co-occurring chronic health conditions [2,3,4]. Multimorbidity refers to patients with two or more coexisting chronic conditions. Conditions include those with a high base rate in older populations, such as cardiovascular risk factors (e.g., hypertension and hyperlipidemia) and musculoskeletal conditions (e.g., arthritis), as well conditions (such as cancer and psychiatric disorders) with a lower geriatric incidence. A study examining the prevalence of multimorbidity across twenty-eight countries indicated that lower socioeconomic status (SES) is associated with an increased risk of multimorbidity [5] and more limited access to health care [6]. There are limited models of care for people with multimorbidity, but the understanding is that “consultation, coordination, and collaboration” are the key for primary care physicians to provide support to the complex service needs of a growing multimorbid population [7].

From a neuropsychological perspective, multimorbidity is poorly understood. While we know relatively little about its effects on cognitive functioning, multimorbidity is associated with greater disability and accelerated decline in physical functioning. Multimorbidity has been considered a predictor of significant functional difficulties in patients with normal scores on a global cognitive screener (MMSE) but not in patients with impaired scores on the same screener [8]. Furthermore, cognitive impairment has also been strongly correlated with the burden of multimorbidity [9,10]. Older patients with multimorbidity, particularly those with neurocognitive disorders, have a higher medical service utilization that results in higher costs, particularly when multidisciplinary care is not integrated into the patient’s treatment plan [9]; yet, these patients respond positively to a multidisciplinary approach to their care, which reduces mortality, increases medication adherence, and cuts medical expenditures [11].

Research on multimorbidity (and the importance of multidisciplinary care) has implications for the broader healthcare system. Multidisciplinary models of care should be considered the gold standard [12]. Furthermore, culturally sensitive care is crucial. Much of the medical care provided in the US is rooted in a Eurocentric perspective that can contribute to the exclusion of minoritized communities, such as those with racial/ethnic and linguistic differences [13]. This can lead to higher rates of misdiagnosis in vulnerable communities, such as older adults of color with multimorbidity. Moreover, cultural sensitivity is of utmost importance when diagnosing or treating a cognitively compromised bilingual individual to ensure instrument appropriateness and assessment validity [14].

The Hispanic paradox suggests that Latinos have a higher life expectancy than their non-Hispanic White and Black counterparts [15], yet they tend to accumulate multimorbidity at a faster rate than their non-Hispanic white counterparts [16]. Language, race, ethnicity, and gender all help to predict the quality of care a patient receives [1]. For example, Hispanic adult patients report more negative patient-centered provider communication than English-dominant/Caucasian respondents [1,17]. When Hispanic patients receive language-concordant care, it can improve their health status [17,18]. Given these barriers and their high rates of multimorbidity, Latino patients can benefit from being served in an integrative, multidisciplinary, and culturally competent healthcare system.

There are many distinctive challenges when providing culturally competent neuropsychological assessments to Spanish-speaking patients [19]. For example, limited normative data can lead to invalid estimates of patients’ mental abilities. False negative cognitive assessment results can lead to harmful outcomes, such as a delay in diagnosis and missed opportunities for timely access to treatment. False positives may cause unnecessary referrals, which affect patients, families, and health services. Given that Latinos are a highly heterogeneous group, culturally competent neuropsychological care is a critical aspect of the comprehensive assessment of Latinos with multimorbidity.

Here, we present a case study of a 69-year-old, left-handed, Hispanic Carribean, cisgender male with 10 years of education. This case was representative of the population we served and illustrated the importance of complex multicultural multimorbid presentations in neuropsychological care, and how they can be managed to create generally favorable outcomes for patients and their families.

## 2. Context and Setting

The UCLA Hispanic Neuropsychiatric Center of Excellence (HNCE) provides interdisciplinary healthcare to the Latino community in Southern and Central California. The center’s focus is to develop models of clinical service, training, and research focused on culturally/linguistically congruent and socially responsible care to minimize diagnostic and treatment disparities in the Latino communities [20]. The HNCE provides a broad spectrum of integrated clinical services, including psychiatric evaluation, psychotherapy, pharmacotherapy, case management, and neuropsychological assessment for socioeconomically diverse patients. The center receives referrals from UCLA specialties, non-UCLA providers, and the LA County Department of Mental Health. The institution is committed to teaching trainees to provide equitable and socially just healthcare services to culturally diverse Spanish-speaking individuals (of ages 4 and above) and their families. As in the case study presented below, the HNCE is often tasked with addressing cognitive functioning in patients with multimorbidity.

In this case study, we described a 69-year-old, Caribbean, bilingual (predominantly Spanish-speaking), married, cisgender male with a complex medical history, including lymphoma, end-stage renal disease, chronic pain, deep vein thrombosis, right internal capsule thalamic stroke, hypertension, thyroid disease, suspected hearing loss, and neuropathy. He was referred by his primary care provider due to concerns with cognitive and emotional functioning.

This patient’s condition could be described as complex multimorbidity. Harrison and colleagues defined this as the “co-occurrence of three or more chronic conditions affecting three or more different body systems within one person without defining an index chronic condition” [21] (p. 9). The present patient’s history of co-occurring conditions, stroke, cardiovascular burden, renal disease, thyroid, neuropathy, and cancer could be described as complex multimorbidity.

Multimorbidity can also be measured based on the co-occurrence of chronic conditions, functional limitations, and/or geriatric syndromes [9]. A patient’s multimorbidity classification could then be coded as MM0 (no chronic conditions, functional limitations, or geriatric syndromes); MM1 (occurrence but no co-occurrence of chronic conditions, functional limitations, or geriatric syndromes); MM2 (co-occurrence of any two of chronic conditions, functional limitations, or geriatric syndromes); and MM3, (co-occurrence of all three of chronic conditions, functional limitations, and geriatric syndromes) [9].

Consistent with Koroukian and associate’s model [9], the present patient would be classified as an “MM3”. The patient reported to experience more than three concurrent chronic health conditions, as previously mentioned. His reported imitations in lower body functioning and strength prevent him from driving a vehicle, therefore impacting his day-to-day functioning. Lastly, he has a self-perceived geriatric syndrome due to suspected non-age-related cognitive decline. That is, he reported that he has had difficulty recalling verbal information and events since 2018. He also has a longstanding history of other cognitive concerns including distractibility, inattention, and executive functioning difficulties. Furthermore, the patient reported being the primary caregiver of his mother who has advanced-stage Alzheimer’s disease.

In 2018, the patient had chemo-refractory lymphoma. He was reportedly told that there were no additional available cancer treatments for him, with medical experts expecting him to have same-year mortality. Since then, he has been stable on a chemotherapy clinical trial, with positive results (including remission as of late 2019) as confirmed by his medical records. Furthermore, after a stroke in 2018, he received post-acute rehabilitative services. This included physical therapy to assist with balance and fall prevention, occupational therapy for daily living and transfer training, speech therapy, and cognitive rehabilitation.

**Figure 1:**
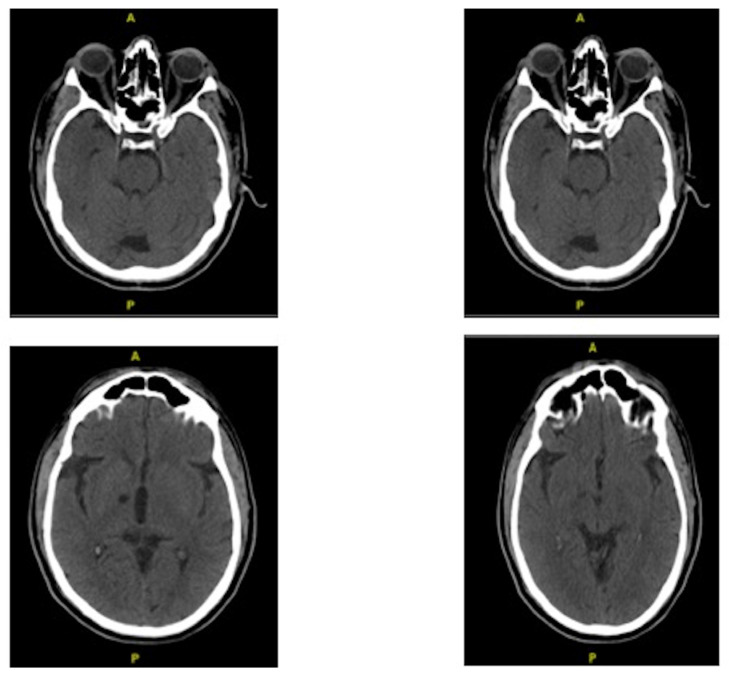
CT Scan results with four cuts in axial view.

Initial neurobehavioral and neurocognitive documentation showed that he was alert and oriented but revealed difficulties with attention, executive control, and memory. A 2020 CT scan of his brain without contrast indicated that there was no evidence of acute intracranial hemorrhage, hydrocephalus, or mass effect. Resolution of a small area of vasogenic edema in the left gyrus rectus that was seen on prior CT scans was noted (see Figure 1). Old lacunar infarcts were observed in the right thalamocapsular region and right cerebellum. Relevant laboratory studies completed four months prior to his neuropsychological evaluation were notable for low white blood cell count, red blood cell count, hemoglobin, hematocrit, MCH concentration, mean platelet volume, neutrophil percent auto, absolute neut count, and immunoglobin. Lab results also included remarkably high B2 microglobulin, alkaline phosphatase, creatinine, total CO_2_, monocyte percent auto, red cell distribution width-CV, mean corpuscular volume, and red cell distribution width-CV.

## 3. Method

Our neuropsychological consultation service considers a patient’s primary language and cultural background and tailors specific neuropsychological testing batteries in the patient’s dominant language using appropriately available linguistic and culturally norms. Test selection is driven by evidence-based practices in clinical neuropsychology for Spanish-speaking communities.

Our approach takes into account ethical and professional factors anchored in the Ethical Code of the American Psychological Association [22,23], including Principle A (Beneficence and Nonmaleficence) [22,23], Principle B (Fidelity and Responsibility; APA, [22]), and Principle D (Justice) [22].

Shelter-in-place orders prevented in-person assessments during portions of the COVID-19 pandemic. The HNCE’s clinical decisions were informed by a comprehensive assessment of the risk associated with delaying neuropsychological evaluation, the need for continuity of care, and a patient’s individual characteristics and medical history [19,24].

Our justification for how we approached the present assessment was as follows:

1. Telehealth: consistent with recommendations established by Arias and colleagues [19], it was determined that teleNP was a valid and appropriate platform to deliver testing to this patient given his multiple health risk factors. Neuropsychologists working at our center have significant experience providing culturally informed services to linguistically diverse patients transferable to remote practices. We were uniquely equipped to develop and implement TeleNP protocols for this population because we had experience integrating clinical observations and cultural factors in order to inform clinical care [19].
2. Language dominance: during clinical interview, it was evident that the patient had minimal expressive and receptive proficiency in English as he was unable to communicate in that language. In fact, he reported Spanish being his preferred language due to growing up in a Spanish-speaking Caribbean country. Most Spanish-language tests selected (e.g., Neuropsi atención y memoria, Batería neuropsicológica en español, etc.) had demographically corrected norms for age, education, and/or gender. However, self-reported measures and one visuospatial test (Judgment of Line Orientation) lacked norms matching the patient’s background. Finding test norms that fit a patient’s demographics is crucial to ascertain appropriate sensitivity and specificity in making a neurocognitive diagnosis.
3. Flexible battery: the battery was developed to evaluate all major neurocognitive domains. All tests and subtests were selected based on the normative reference group considering language, age, education, country of origin, and gender. The battery selected included the following:
  - Batería III Woodcock-Muñoz pruebas de aprovechamiento [25], which is an academic achievement measure that has been culturally adapted from the Woodcock Johnson III Test of Achievement. One subtest (Letter-Word Identification) was selected to aid in the estimation of the patient’s educational level and premorbid cognitive functioning.
  - Batería neuropsicológica en español (BNE) [26], which is a neuropsychological battery developed in Spanish that uses a combination of age-matched norms (18–65 years of age), education-matched norms (0–16 years-of-education), and country of origin (Spain and Mexico/US border) norms. Specific subtests used in this evaluation included auditory attention and auditory working memory (BNE Atención verbal/Digit Span), verbal processing speed and inhibition (BNE Prueba Stroop/Stroop Test), phonemic verbal fluency (BNE Fluidez oral por letra/Phonemic Fluency), and verbal memory in context (BNE Memoria verbal-prosa/Logical Memory).
  - The Dot Counting Test [27], Rey-15 Item Test [28], and Test of Memory Malingering (TOMM) [29] were measures selected to assist with verifying the patient’s test-taking effort.
  - The Geriatric Anxiety Inventory, Spanish Version (GAI) [30] was selected to measure the patient’s anxiety symptomatology and to aid in possible clinical syndrome diagnosis.
  - Judgment of Line Orientation [31], which is a test that measures visuospatial perception and orientation. The test considers score corrections for age and gender but not for race/ethnicity, education, or other demographic factors.
  - Millon Behavioral Medicine Diagnostic, Spanish version (MBMD) [32] was selected to assist in identifying psychosocial factors that might contribute to the patient’s medical condition and course of treatment.
  - Neuropsi atención y memoria [33,34,35], which is a neuropsychological battery developed in Spanish with age-matched norms (6–85 years-of-age), education-matched norms (0–22 years-of-education), and ongoing norm and reference group publications (e.g., NP-NUMBRS project) [36]. Selected subtests for the present assessment included the Rey–Osterreith Complex Figure Test, Semantic Fluency, and Motor Programming.
  - Neuropsychological Screening Battery for Hispanics (NeSBHis) [37], which is a neuropsychological assessment battery with norms by age (16–75 years-of-age), education (lower than vs. higher than 10 years of education), and gender. Selected subtests for this evaluation included a confrontation naming task (Pontón–Satz Boston Naming Test), a verbal memory task (WHO-UCLA Auditory Verbal Learning Test), and a graphomotor processing speed task (EIWA Digit Symbol test).
  - The Spanish Geriatric Depression Scale (GDS] [38] was selected to measure depressive symptomatology and to aid in possible clinical syndrome diagnosis.
  - The Woodcock-Johnson IV Tests of Oral Language (TOL) [39], which can be used to help determine a patient’s expressive/receptive language functioning in Spanish and/or English. For the present assessment, expressive (Vocabulario sobre dibujos) and receptive (Comprensión oral) subtests were administered in Spanish.
4. Culturally sensitive adaptive functioning assessment: a collateral informant reported that as a result of the patient’s initial lymphoma diagnosis in 2018 and refractory chemotherapy treatment, his everyday functioning changed, primarily impacting scheduling and driving. Schedule-wise, the patient reportedly used to manage his own schedule, including his medical appointments and medications. After chemotherapy treatment, the patient has been reportedly unable to manage his medical appointments. For medications, the patient’s wife had to set up alarms on the patient’s phone to remind him to take his medications, though the patient still forgets to check his phone on multiple occasions. In terms of driving, the patient self-reported that he is able to drive; however, due to his balance and motor changes post-stroke, family members do not feel comfortable with him driving by himself. The patient is independent in all basic activities of daily living. Regarding instrumental activities of daily living (IADLs), he is generally dependent on others for transportation, medication, and money management. He further explained that he has never managed his finances, as his wife took the initiative to do these early on in their relationship, which she reported was culturally normative for them. He can also prepare simple meals, uses his mobile phone, reads for entertainment, shops, and performs daily housekeeping tasks with none-to-minimal difficulties.

## 4. Results

The present patient is one with complex multimorbidity. Our comprehensive neurocognitive evaluation conducted in 2020 via telehealth shed light onto the patient’s strengths and weaknesses using culturally and linguistically tailored measures (see Table 1 for neurocognitive results). The patient and his family reported serious concerns for memory deficits, which may have been erroneously measured if the assessment were completed in English or with a Spanish–English interpreter.

Instead, key results from our multicultural assessment included the following. (A) The patient had generally preserved expressive and receptive language abilities when compared to same-aged peers with similar levels of education. His premorbid functioning was estimated to be at least within the average range, based on his level of education, his occupational trajectory, and objective testing of his reading in Spanish (notably, his scores in Spanish word reading were not directly used to determine his premorbid functioning, as the Spanish language is more of a transparent orthographical language compared to English). (B) The patient had a well-preserved verbal memory. (C) The patient had impaired visuoconstructional abilities that impacted his nonverbal encoding and graphomotor processing speed. These visuoconstructional deficits were consistent with the sustained cerebrovascular lesions in the thalamocapsular and right cerebellar regions, which likely affected sensory integration. (D) The patient also had increased irritability, crying, and sadness, which might in part be due to findings of mass effect on the posterior left gyrus rectus (despite an apparent resolution of these findings in his February 2020 CT scan).

Although his performance was within normal limits for monolingual individuals on a task of inhibition, studies have shown stronger performance on such tasks in bilingual individuals [40]; thus, the present patient was described as having relative difficulties in inhibition. These were consistent with his labile behavioral presentation, underlying cerebrovascular pathology, and likely historical concern with executive functioning. Further findings such as variable attention, visuoconstructional skills, and graphomotor processing speed were also aligned with his medical conditions (i.e., stroke in the thalamocapuslar region, right cerebellum lesions, cardiovascular burden). Similarly, mood symptoms were attributed in part to the mass effect on the posterior left gyrus rectus.

## 5. Discussion

Multimorbidity is extremely prevalent in the older Latino community [16,41]. Older adults with multimorbidity present with both neurocognitive and functional limitations. These conditions require an interdisciplinary diagnostic workup, polymedication management, and interagency collaboration. The need for an integrative, multidisciplinary, and culturally sensitive model of care to support the complex needs of diverse patients with multimorbidity is imperative. While limited, several studies have observed that a multidisciplinary approach is effective at optimizing patients’ health care and longevity [42,43,44]. Not only is multimorbidity a predictor of disability and loss of independence regarding activities of daily living (ADLs), but cognitive functioning also predicts the development of disability and decline in ADLs in older populations [8].

The present case involved a complex presentation of cognitive complaints that reportedly limited the patient’s daily activities, and consequently, impacted his life and his family. He was also expected to have a short-term mortality due to his cancer while caring for his mother with Alzheimer’s disease, which added to his levels of stress.

Under our center’s model of care, the patient was assessed, taking into consideration his primary language, his cultural-psychosocial background, and lived experiences. Culturally and linguistically congruent norms were used to interpret his neurocognitive profile. An estimate of his general education was important to determine the expected performance to compare his current results. To this end, it was important to collect specific demographic background, including primary language of his formal education, level of bilingualism, and occupation. Within this context, our patient was found to have preserved linguistic skills, and his verbal memory presented no difficulties at the levels of encoding, storage, or retrieval. His visual memory was impaired; however, his visuoconstructional skills were variable, which was possibly due in part to a visual deficit for which he was referred to neuro-ophthalmology for further rule-out. Due to the patient’s cardiovascular risk factors, cancer, metabolic conditions, and his current functional abilities, his final diagnosis was mild Neurocognitive Disorder due to Multiple Etiologies.

This case benefited from being presented to our center’s weekly multidisciplinary group case conference, where input from multiple professionals, including consultation with a neurologist, facilitated the understanding of the etiology of his neurocognitive and mood symptoms and helped tease out the possible role of psychological vs. neurological factors. Consultation with neurology also improved the integration of neuroimaging and neuropsychological results. We also consulted with our center’s social work team, and specific recommendations were developed to respond to the patient and his family’s needs in concordance with our social equity model. Similarly, our feedback session was crucial, not only to communicate results in a sensitive manner, but also to answer all questions, foster connection, and ensure understanding, agreement, and adherence to the treatment plan. The inclusion of social workers in the multidisciplinary team facilitated continuity of care by improving communication with other physicians and coordinating integrated services. For example, in the present case, the patient and his family benefited from assistance in scheduling follow-up visits with neuro-ophthalmology, audiology, and family therapy (through our university’s Spanish-Speaking Psychosocial Clinic) to process his new role in the household and provide tools and support to the patient and his family.

In summary, this case illustrated the importance of culturally competent neurocognitive assessment and the advantages of a multidisciplinary approach to health care, with close communication among health care professionals to facilitate diagnostic accuracy, tailor recommendations, and ensure treatment adherence. It also supported existing evidence about the benefits of multidisciplinary work in handling multimorbidity. To this end, our center is developing a health system approach that responds to specific needs, and involves multidisciplinary care, where collaboration, coordination, and communication are crucial.

## Figures and Tables

**Table 1:** Neuropsychological data results.

Performance Validity Testing	Raw Score	Rating
Dot Counting Test E-Score [27]	11	Acceptable
Rey-15 Item Test [28]	21	Acceptable
TOMM Trial 1 [29]	50	Acceptable
TOMM Trial 2 [29]	50	Acceptable
Academic Achievement	Raw Score	Standard Score
Batería III Letter-Word ID (GE > 18.0) [25]	74	(Age) 113
		(Grade) 132
Attention/Working Memory	Raw Score	Standard Score
BNE Digit Span (BNE-DS) [26]		(T-score)
DS forward (LS = 4)	4	32
DS backwards (LS = 5)	5	61
Processing Speed		
BNE Stroop Test [26]	Raw Score	Standard Scores
Word reading total	102	57
Color naming total	68	58
EIWA Digit Symbol [37]	24	Std = 77
Language	Raw Score	Standard Score
Pontons BNT total correct (+0 pt. w/phonemic cues) [37]	25	Z = −0.85
Verbal Fluency (Spanish)		
BNE letter fluency (P:11, M:9, R:11) [26]	31	T = 48
NEUROPSI A&M semantic (animals) Fluency (P = 0, I = 0) [35]	19	Z = 0.13
Test of Oral Language [39]		
Vocabulario sobre dibujos	39	SS = 91
Comprensión oral	29	SS = 92
Visuospatial Functioning	Raw Score	Standard Score
NEUROPSI A&M RCFT-Copy [35]	27	Z = −2.26
Judgement of Line Orientation (+3 age correction) [31]	21	%ile = 40
Verbal Memory	Raw Score	Standard Score
WHO-UCLA AVLT [37]		(z)
Total Trial I-V learning curve	6/12/11/14/11	
Trial V (I = 0, P = 0)	11	−0.10
Trial VII—short delay (I = 1, P = 0)	8	−0.30
Trial VIII—long delay (I = 1, P = 3)	9	−0.37
Trial IX—identification	14	
BNE Logical Memory [26]		(T)
Trial 1	10.5	55
Learning score 10.5/19	9.50	61
Long delay recall	19.5	72
% retention	103	
% recognition discriminability	94	62
Hits:	16	FP: 0
Non-Verbal Memory	Raw Score	Standard Score
NEUROPSI A&M RCFT [35]		(z)
Long delay recall	8	−1.10
% retention	30%	
BVMT-R [36]		(T)
Total immediate recall 1/3/4	8	36
Delayed recall	4	46
% retention	100%	
Hits:	6	FP: 3
Discrimination index	3	
Response bias	0.88	
Executive Function	Raw Score	Standard Score
BNE Stroop Test [26]		(T)
Color/word	27	53
Predicted	41	61
WCST [26]		(T)
Trials administered	128	50
Total correct	96	86
Total errors	32	69
Perseverative responses	13	64
Perseverative errors	13	
Nonperseverative errors	19	
Conceptual level responses	88	
Categories completed	5	62
Trials to complete 1st category	24	
Failure to maintain set	3	
Learning to learn	−3.44	
Motor Functions	Raw Score	
NEUROPSI A&M Motor Functions [35]		
Opposite reactions	2/2	
Inhibitory reactions (Go/No-Go)	2/2	
Motor programming (RH)	2/2	
Motor programming (LH)	2/2	
Psychosocial Functioning	Raw Score	Standard Score
Geriatric Depression Scale [38]	15	
Geriatric Anxiety Inventory [30]	3	
MBMD [32]		
Anxiety-tension	8	61
Depression	12	73
Cognitive dysfunction	12	63
Emotional lability	4	35
Guardedness	18	70
Introversive	14	84
Inhibited	5	64
Dejected	3	60
Cooperative	18	92
Sociable	12	58
Confident	12	55
Nonconforming	14	62
Forceful	12	54
Respectful	22	61
Oppositional	16	71
Denigrated	2	35
Illness apprehension	19	75
Functional deficits	22	87
Pain sensitivity	27	95
Social isolation	7	61
Future pessimism	15	72
Spiritual absence	21	90
Interventional fragility	7	42
Medication abuse	6	64
Information discomfort	0	0
Utilization excess	8	62
Problematic compliance	7	52
Adjustment difficulties	6	65
Psych referral	6	70

## Data Availability

The clinical data obtained and used in this case report has been made available in the body of the manuscript. However, specific details including raw data or item-level responses have not been made available due to ethical and legal restrictions.
